# Association between formal thought disorders, neurocognition and functioning in the early stages of psychosis: a systematic review of the last half-century studies

**DOI:** 10.1007/s00406-021-01295-3

**Published:** 2021-07-14

**Authors:** Oemer Faruk Oeztuerk, Alessandro Pigoni, Linda A. Antonucci, Nikolaos Koutsouleris

**Affiliations:** 1 Department of Psychiatry and Psychotherapy, Ludwig-Maximilian-University Munich, Nussbaumstr. 7, 80336 Munich, Germany; 2 International Max Planck Research School for Translational Psychiatry, Munich, Germany; 3 MoMiLab Research Unit, IMT School for Advanced Studies Lucca, Lucca, Italy; 4 Department of Basic Medical Sciences, Neuroscience and Sense Organs–University of Bari “Aldo Moro”, Bari, Italy; 5 Department of Education, Psychology and Communication Science–University of Bari “Aldo Moro”, Bari, Italy; 6 Department of Psychosis Studies, Institute of Psychiatry, Psychology and Neuroscience, King’s College London, London, UK; 7 Max-Planck Institute of Psychiatry, Munich, Germany

**Keywords:** Formal thought disorder, Clinical high risk, Psychosis, Functioning, Neurocognition

## Abstract

Recent review articles provided an extensive collection of studies covering many aspects of format thought disorders (FTD) among their epidemiology and phenomenology, their neurobiological underpinnings, genetics as well as their transdiagnostic prevalence. However, less attention has been paid to the association of FTD with neurocognitive and functioning deficits in the early stages of evolving psychosis. Therefore, this systematic review aims to investigate the state of the art regarding the association between FTD, neurocognition and functioning in the early stages of evolving psychotic disorders in adolescents and young adults, by following the PRISMA flowchart. A total of 106 studies were screened. We included 8 studies due to their reports of associations between FTD measures and functioning outcomes measured with different scales and 7 studies due to their reports of associations between FTD measures and neurocognition. In summary, the main findings of the included studies for functioning outcomes showed that FTD severity predicted poor social functioning, unemployment, relapses, re-hospitalisations, whereas the main findings of the included studies for neurocognition showed correlations between attentional deficits, executive functions and FTD, and highlighted the predictive potential of executive dysfunctions for sustained FTD. Further studies in upcoming years taking advantage of the acceleration in computational psychiatry would allow researchers to re-investigate the clinical importance of FTD and their role in the transition from at-risk to full-blown psychosis conditions. Employing automated computer-assisted diagnostic tools in the early stages of psychosis might open new avenues to develop targeted neuropsychotherapeutics specific to FTD.

**Supplementary Information:**

The online version contains supplementary material available at 10.1007/s00406-021-01295-3.

## Introduction

Formal thought disorders (FTD) are psychopathological alterations that can emerge in different psychiatric disorders such as schizophrenia, major depressive disorder, and mania. [1, 2] FTD are a multidimensional construct involving thought, language, and communication disturbances, such as loosening of associations, blocking, semantic and phonemic paraphasia. [1, 3] It has taken one and half centuries to formulate FTD as such a construct, starting from Esquirol pointing to a primary pathology in coordinating ideas [4] (1838) and from Griesinger’s differentiation [5] between “formal deviations” and “false contents” (1867). Further clinical developments over the twentieth century led to further conceptualizations of FTD, such as “dementia praecox” [6] from Kraepelin (1919), “derailment” [7] from Schneider (1930), “concrete thinking” [8] from Goldstein (1944), “loosening of associations” [9] from Bleuler’s (1950), and Andreasen’s (1985) revision of FTD as a multidimensional psychopathological construct of disturbances in thought, language and communication [10]. (Jerónimo et al*.* 2018 for more details [11]).

FTD severity has been assessed with different scales since Kraepelin and Bleuler postulated the importance of earlier manifestation of this clinical phenomenon in an evolving psychosis [12]. However, before the development of specific scales such as—in chronological order—Andreasen’s scale for the assessment of thought, language and communication (TLC) [10], the Thought and Language Index (TLI) [13], the Thought Disorder Index (TDI) [14], and the scale for the assessment of Thought, Language and Disorder (TALD) [3], FTD severity has been assessed through items part of non-FTD-specific psychopathological scales, such as the Positive and Negative Syndrome Scale (PANSS) [15], the Scale for the Assessment of Positive Symptoms (SAPS) [16], the Scale for the Assessment of Negative Symptoms (SANS) [17] and the Brief Psychiatric Rating Scale (BPRS) [18]. These non-specific scales usually address one or only a few psychopathological aspects of FTD. [2] Moreover, the heterogeneity [2] of the specific scales makes quantitative comparison of findings in literature difficult, as they capture various psychopathological aspects of FTD.

Recent review articles provided an extensive collection of studies covering many aspects of FTD among their epidemiology and phenomenology, their neurobiological underpinnings, genetics, their neurological correlates, as well as their transdiagnostic prevalence. [1, 2, 11, 12] However, literature regarding the investigation of FTD as early clinical signs of an evolving psychosis, as well as their prognostic importance, is rather limited. Indeed, the majority of findings addressed FTD relevance for disease course either in chronic patients or in patients with a highly heterogeneous age range (18–65). [19, 20] The limited literature in early stages of psychosis revealed FTD not only as a core feature of psychosis but also in association with several psychosis-related adverse outcomes, such as functional impairments and cognitive deficits. [2, 3, 11, 12, 21] These findings are particularly relevant, as functional and neurocognitive impairments [22, 23] frequently precede disease onset and persist after remission of the acute illness in psychotic disorders. [24, 25]. Consistently, given perspective changes and contributions in the last decades regarding early diagnostic tools aimed at recognizing such persisting impairments with an impact on real-world prognostic outcomes, research focus has been turned on risk groups and younger patients experiencing first-episode psychosis. [26–29] With specific respect to FTD, only very few studies have highlighted that their severity has been associated with increased (re-)hospitalization rate [19], unemployment risk [30] and reduced quality of life [31]. Moreover, patients with schizophrenia experiencing enduring FTD after the acute phase of psychosis showed lower occupational functioning levels and higher relapse rates. [32] On the other hand, FTD have been associated with attentional executive functions deficits at the early stages of psychosis, [33, 34], even if this association has not been fully replicated yet. Furthermore, executive dysfunctions at the early stages of psychosis seem to predict FTD severity at follow up [35]. However, despite these findings and the increasing interest in early diagnostics and prevention in psychotic disorders, literature on prodromal state and first-episode psychosis so far did not provide any target for preventive interventions based on the core psychopathological changes such as FTD predicting adversity in clinical outcomes, yet. A potential way to understand this is to thoroughly investigate whether and how FTD are related to relevant outcomes of psychosis, i.e., deficits already present in their early stages, such as functioning and neurocognitive impairments.

Therefore, we conducted a systematic review regarding the state of the art of the association between FTD, functioning outcomes and neurocognitive impairments in the early and prodromal stages of psychotic disorders. We summarized the main findings of the included studies providing evidence related to the early diagnostic and prognostic potential of FTD in association with functioning and neurocognition. Furthermore, we discussed possible reasons for the paucity of studies investigating the clinical relevance of FTD in early psychosis and presented new perspectives for potential future investigations on FTD with the help of modern computational analytical techniques that might serve as computer-assisted early diagnostic tools. We concluded with an outlook for future research targeting the potential preventive and predictive role of FTD in psychosis trajectories.

## Methods

We conducted two separate systematic literature searches; (i) association between FTD and functioning outcomes and (ii) association between FTD and neurocognition, both in the early and prodromal stages of psychosis, by following the Preferred Reporting Items for Systematic Reviews and Meta-Analyses (PRISMA) [36] statement. Two of the authors (Ö.F.Ö and A.P.) independently conducted the systematic literature searches in PubMed, PsychINFO and Web of Science covering the last half-century until 2019.

For the literature search regarding the association between FTD and functioning outcomes, we used the following search terms combination: (“formal thought disorder” OR “thought disorder”) AND (child OR adolescent OR young adult) AND (psychosis OR “early-onset schizophrenia” OR “first episode psychosis” OR “recent onset” OR “high risk” OR prodrom) AND (“GAF” OR “GF” OR “functioning” OR “disability” OR occupational OR social. For the literature search regarding the association between FTD and neurocognition, we used the following search terms combination: (“formal thought disorder” OR “thought disorder”) AND (child OR adolescent OR young adult) AND (psychosis OR “early onset schizophrenia” OR “first episode psychosis” OR “recent onset” OR “high risk” OR prodrom) AND (neurocognition OR neurocognitive or cognition or cognitive or neuropsycho OR memory OR executi OR process).

Articles had to meet the following inclusion criteria: (1) reported statistically significant associations between FTD and either functioning outcomes, or neurocognitive measures; (2) did include high-risk groups [37] such as ultra-high risk (UHR), attenuated psychotic symptoms (APS), the brief limited intermittent psychotic episode (BLIPS) and genetic risk and deterioration syndrome (GRD) or prodromal phases or early stage of psychosis such as first-episode psychosis (FEP), early psychosis (EP) (Onset of Psychosis < 2 years); (3) did include children, adolescents or young adults (< 35 years of age); (4) did include data of functioning outcomes or neurocognition. Articles were excluded for the following reasons: (a) not in English, (b) sample composed only of participants with chronic psychosis or with an onset of disease > 2 years (c) sample composed only of adult patients (18–65 age range) without any specific subgroups analyses based on age ranges (d) did include individuals with drug-induced psychosis (e) did include individuals with psychosis due to medical conditions. Case series, book chapters, literature reviews, conference papers, meeting abstracts or meta-analyses were also excluded.

Based on these criteria, as a first step, two authors have screened the titles and abstracts separately in three different databases (PsycINFO, Medline and Web of Science), and the results of included and excluded articles were discussed among co-authors. Based on the abstracts screening, records for which an inclusion or exclusion decision could not be taken were listed. Thus, in a second round, the same two authors have screened the full texts of these articles to check their eligibility for inclusion. Records for which both authors agreed on their inclusion were then proved for their eligibility again through further full-text investigation.

**Fig. 1:**
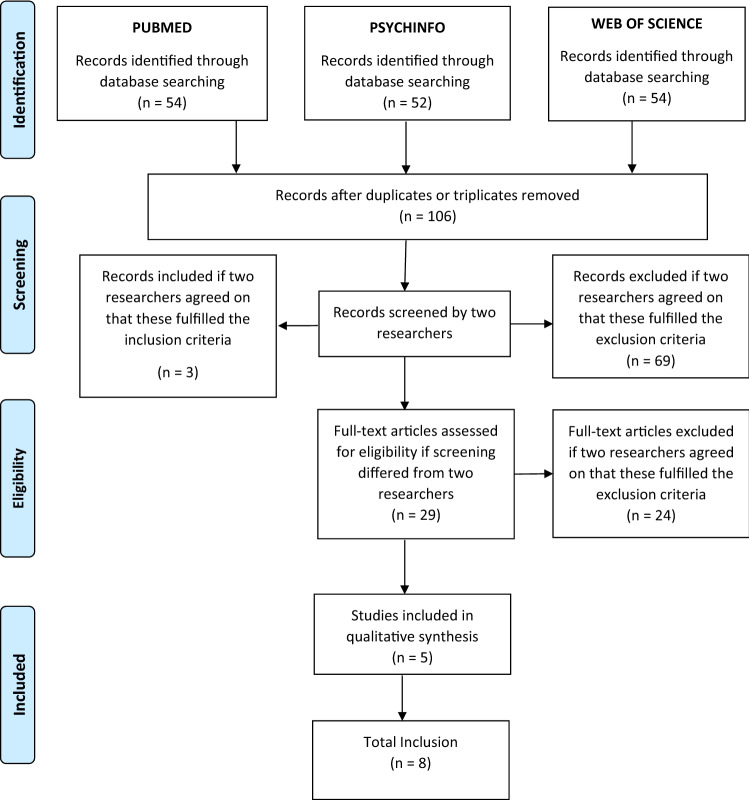
Prisma Graphs representing the inclusion of the studies related to functioning and FTD association

**Fig. 2:**
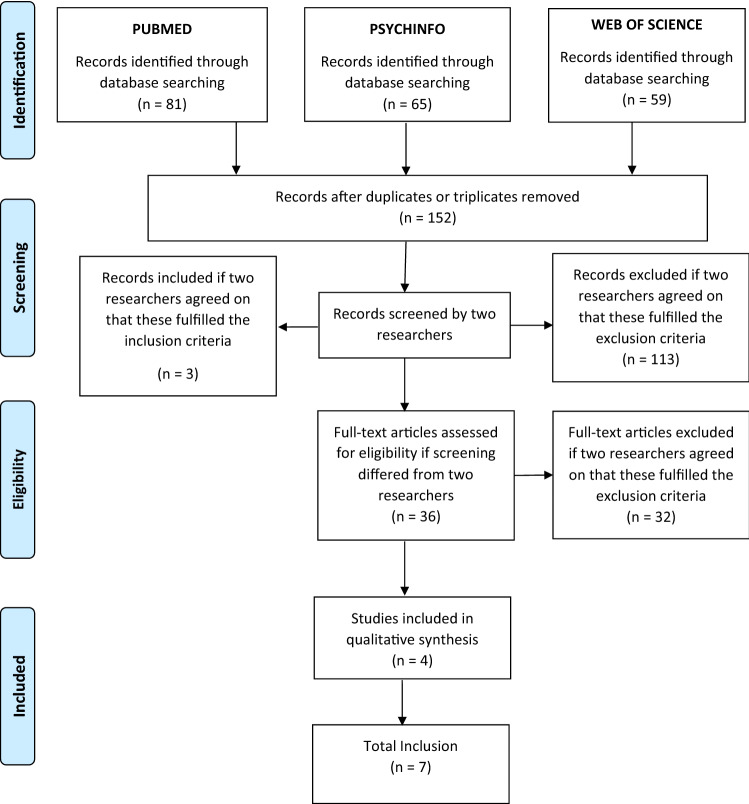
Prisma Graphs representing the inclusion of the studies related to neurocognition and FTD association

The procedure used for the selection of studies for each literature search is reported in Figs. 1 and 2. The included studies are summarized in Tables 1 and 2.

**Table 1:** A list of included studies for associations between FTD and functioning outcomes

First author	Publication year	Published journal	Study groups	Sample size	Mean age	FTD measures	Outcome measures	Main findings and conclusion
Harrow, M	1986	Schizophrenia Bulletin	SCZ, other psychotic patients, and non-psychotic patients	191	23	Bizarre-idiosyncratic thinking	Occupational functioning, subsequent unemployment, relapse/re-hospitalisation rates (LKPS and SCS)	Subsequent unemployment and higher relapse/re-hospitalisation rates in schizophrenia patients experiencing enduring FTD
Marengo, JT	1987	Arch Gen Psychiatry	SCZ, other psychotic, and non-psychotic patients	191	23	Bizarre-idiosyncratic thinking	Occupational functioning, subsequent unemployment, relapse/re-hospitalisation rates (LKPS and SCS)	Patients with enduring thought disorder signs proved to be a poor-outcome group
Racenstein, JM	1999	J Nerv Ment Dis	SCZ, other psychotic, and non-psychotic patients	191	23	Bizarre-idiosyncratic thinking	Occupational functioning, subsequent unemployment, relapse/re-hospitalisation rates (LKPS and SCS)	FTD and functioning correlated in the first eight-year of schizophrenia. A stronger correlation between FTD and occupational than social functioning
Kotov, R	2016	Journal of Abnormal Psychology	First-admission inpatients with psychosis	628	30	SAPS and SANS	GAF, QLS (Social and role functioning), residential independency	A four-factors model (reality distortion, disorganization, inexpressivity, and apathy/asociality) having a stable and replicable validity in predicting outcomes
Minor, KS	2016	J Abnorm Psychol	Early stages of psychosis	38	24.89	CDI	GFS and GFR	Positive FTD and affective reactivity were consistently accounting for poor social functioning and associated with poor role functioning in some cases in EP
Roche, E	2016	Schizophrenia Research	FEP	680	33.42	Disorganization, verbosity, poverty of speech	Social and occupational functioning (MIRECC GAF subscales), number of hospitalisations	Higher baseline severity of disorganization predicted a greater number of hospitalisations and prolonged hospitalisation during the first year of illness
Burton, CZ	2019	Schizophrenia Research	CLR, CHR, EFEP	327	16.69	SIPS	GFS and GFR	Baseline negative symptoms and thought disorder appeared to predict the functional outcome for up to 2 years among adolescents and young adults at risk for psychosis
Bearden, CE	2011	Journal of the American Academy of Child and Adolescent Psychiatry	CHR	105	16.66	Illogical thinking, poverty of content (POC), and referential cohesion	GFS and GFR	Transited to psychosis, predicted significantly social and role functioning at follow-up

*FTD* formal thought disorder, *CDI* communication disturbances index, *FEP* first episode psychosis, *EP* early psychosis, *CLR* clinical low risk, *CHR* clinical high risk, *UHR* ultra-high risk, *HR* high risk, *LR* low risk, *ARMS* at-risk mental state, *APS* attenuated psychosis syndrome, *APSS* attenuated positive symptom syndrome, *LKPS* levenstein, klein, and pollack scale, *SCS* strauss and carpenter scale, *OPCRIT* operational criteria for psychotic illness tool, *SAPS* scale for the assessment of positive symptoms, *SANS* scale for the assessment of negative symptoms, *CAARMS* comprehensive assessment of at-risk mental states, *SIPS* structured interview for prodromal syndromes, *SCZ* schizophrenia, *SOPS* scale of prodromal symptoms, *WERCAP* Washington early recognition center affectivity and psychosis screen, *GAF* global assessment of functioning, *GFS* global functioning scale-social *GFR* global functioning scale-role, *QLS* quality of life scale

**Table 2:** A list of included studies for associations between FTD and neurocognition

First author	Publication year	Published journal	Study groups	Sample size	Mean age	FTD measures	Outcome measures	Main findings and conclusion
Minor, KS	2016	J Abnorm Psychol	EP	38	24.89	CDI	Single- and dual-task one-back visual working, memory tests	Affective, but not cognitive, systems play a critical role in positive FTD
Nuechterlein, KH	1986	Schizophrenia Bulletin	Early phase of SSD	32	22.3	The Rorschach TDI, the BPRS conceptual disorganization rating	CPT and forced-choice span of apprehension	The only significant correlations of the outpatient signal discrimination indices with inpatient positive symptoms were with conceptual disorganization
Xu, Jia-Qi	2014	Schizophrenia research	First-episode, SSD	60	25.28	CLANG	HSCT, MCT, modified SET, LNST, modified WCST	Poorer performances in sustained attention and attention allocation/planning at illness onset were associated with an increased risk of having residual semantic levels of language disorganization after one year
Pawelczyk, A	2018	Psychiatry research	FEP, HC, parents of FEP	34, 34, 32	20.85, 20.21, 49.44	The right, hemisphere language battery	TMT part A, TMT part B, DST–B, DST-F	Pragmatic dysfunctions may act as vulnerability markers of schizophrenia
Caplan, R	1992	Journal of the American academy of child and adolescent psychiatry	SCZ	31	10.2	The kiddie formal thought disorder rating scale	The Wechsler intelligence test for children revised	Lexical cohesion correlated negatively with full-scale IQ and performance IQ scores but not with the verbal IQ scores in the patients with loose of associations
Remberk, B	2012	Progress in Neuro-Psycho, pharmacology and Biological Psychiatry	SSD, HC	32, 32	16.7, 16.7	TLC, KRT	WCST, SVFT, PVFT, DST-B, DST-F	Thought disorder was correlated with executive dysfunction and disturbance in semantic verbal fluency
Ilonen, T	2010	Psychiatry research	CHR, psychotics, non-psychotic/non-CHR individuals	22, 67, 187	15.6, 15.7, 15.5	The Rorschach, PTI	Verbal comprehension, perceptual organisation, working memory, processing speed, executive function, perceptual and thinking accuracy	The deficits were comparable in severity to those observed in adolescents with psychotic diagnoses and that patients at CHR for psychosis displayed mild-to-moderate executive impairment, without any impairment in intellectual functioning

*FTD* formal thought disorder, *CDI* communication disturbances index, *EP* early-stage psychosis, *FEP* first-episode psychosis, *CHR* clinical high risk, *HC* healthy control, *CPT* continuous performance test, *SCZ* schizophrenia, *SSD* schizophrenic spectrum disorders, *TDI* thought disorder index, *TLC* thought, language and communication scale, BPRS brief psychiatric rating scale, *CLANG* clinical language disorder rating scale, PTI perceptual thinking index, *KRT* Kent–Rosanoff test, *HSCT* Hayling sentence completion test, *MCT* monotone counting test, *SET* six element test, *LNST* letter number sequence test, WCST Wisconsin card sorting test, *TMT* trail making test, *DST*-*B* digit span test backward, *DST*-*F* digit span test forward, *SVFT* semantic verbal fluency test, *PVFT* phonological verbal fluency test

## Results

### Functioning outcomes and FTD

We screened 106 studies in total and included 8 of them. These studies were included due to the presence of significant associations between FTD in adolescents or young adults and their reports, and functioning outcomes measured with different scales. Overall, the included studied showed that higher severity of FTD predicted poor social functioning, unemployment, relapses, re-hospitalisations. Three studies, namely Harrow. [38], Marengo [30] and Racenstein et al*.* [39], reported results from the Chicago Follow-up Study, assessing the importance of FTD through a comprehensive measure of bizarre-idiosyncratic thinking in the acute phase of inpatients at baseline, and at follow-ups taking place 1 ½, 2, 4 and 8 years after hospital discharge. They reported that approximately 30% of patients diagnosed with schizophrenia exhibited persisting thought disorders during follow-up periods and that such patients with enduring thought disorder signs were also a poor-outcome group. Specifically, they highlighted a poorer prognosis with lower occupational functioning levels (subsequent unemployment 82% and higher relapse/re-hospitalisation rates: psychosis 68% and rehospitalization 59%) in schizophrenia patients experiencing enduring FTD after the acute phase. Moreover, they showed that FTD and functioning levels are correlated in the first 8 year of schizophrenia, with a stronger correlation between FTD and occupational functioning compared with social functioning.

Kotov et al*.* [40] interviewed 628 inpatients with first-episode psychosis six times over two decades in an epidemiologic cohort. They showed that a four-factor model (reality distortion, disorganization, inexpressivity, and apathy/asociality) could significantly predict functional outcomes. Interestingly, they observed that apathy/asociality predicted impairments in global functioning, social functioning, role functioning, and life satisfaction, whereas inexpressivity predicted lower residential independence. Minor et al*.* [41] assessed the positive FTD via the Communication Disturbances Index (CDI) [42] and speech production in early psychosis (EP) individuals and controls, and explored their association to real-world outcomes. They reported large differences in both positive FTD and speech production between EP individuals and controls, and showed that positive FTD and affective reactivity were associated with poor social and role functioning only in EP. Moreover, they found that positive FTD and affective reactivity were consistently accounting for poor social functioning (up to 56% of social functioning’s variance) and associated with poor role functioning (accounting for up to 46% of the variance) in EP.

Roche et al*.* [19] evaluated the relationship between FTD features (namely, disorganization, verbosity and poverty of speech) and social and occupational functioning outcomes at baseline and 1 year later in a first-episode psychosis cohort. This study found that only disorganization was associated with functional outcome, specifically social functioning and that the longitudinal course of disorganization remained significantly associated with social functioning on multivariate analysis. Moreover, they reported that higher baseline severity of disorganization predicted a greater number of hospitalisations and prolonged hospitalisation during the first year of illness. Burton et al*.* [43] tested the efficacy of early intervention for youth at risk of developing psychosis in a multisite national trial dataset, where they followed participants prospectively at 6, 12, and 24 months, and examined the relationships between baseline symptoms and longitudinal global social and role functioning. They found that higher baseline negative symptoms and deteriorated thought process predicted worse social and role functioning for up to 2 years among adolescents and young adults at risk for psychosis, and that the changing effect of negative symptoms on social functioning over time was moderated by positive symptoms. Bearden et al*.* [44] examined the association of baseline FTD assessed by the Kiddie Formal Thought Disorder Rating Scale (K-FTDS) [45] and linguistic cohesion with conversion to psychosis and social and role outcome at follow-up (approximately 1 year later) by analyzing transcribed speech samples in individuals with a clinical high-risk for psychosis. They reported that baseline poverty of content (POC) and referential cohesion predicted significantly social and role functioning at follow-up.

### Neurocognition and FTD

We screened 106 studies in total and included 7 of them. These studies were included due to the existence of significant associations between FTD in adolescents or young adults, and neurocognition. The study from Minor et al. [41] was included both in this section and on the one regarding functioning, as this study investigated the association between FTD and both domains. Overall, the included studies for the associations between FTD and neurocognition showed heterogeneous findings. Still, some consistent findings were observed for what concerns associations between attentional deficits, executive functions and FTD.

Minor et al*.* [41] explored associations between positive FTD assessed by the Communication Disturbances Index (CDI) [42] and speech production assessed by 2 min records about negative (affective condition) and neutral (baseline, cognitive conditions) memories in early psychosis (EP) individuals and controls. They employed a single task for baseline condition, where participants completed only one-back visual working memory test, whereas a dual task for cognitive condition, where participants completed the one-back visual working memory test while simultaneously generating speech. They showed that cognitive reactivity appeared to have a less defined role than affective reactivity in positive FTD. Based on these findings, they suggested that the cognitive load effect may not translate to positive FTD or speech production in early psychosis. In contrast, affective systems played a prominent role in positive FTD that EP individuals exhibited a steeper increase in positive FTD from the baseline to the affective condition compared to controls.

Nuechterlein et al*.* [33] investigated the association between three cognitive tests in the attention domain (namely, two versions of the continuous performance test (CPT) and forced-choice span of apprehension task) and FTD assessed through Brief Psychiatric Rating Scale (BPRS) Conceptual Disorganization subscores, Rorschach Thought Disorder Index factors, and negative symptoms assessed by BPRS Anergia factor scores in inpatients experiencing an early phase of schizophrenic disorders. They retested 32 patients after clinical stabilization to address the extent to which continued attentional deficits were associated with specific symptomatology during the hospitalized period. Their results indicated that signal discrimination deficits were consistently related to the presence of negative symptoms and that the correlation of the attentional deficits to FTD was significant, even though to a less extent. In clinically stabilized outpatients, they reported significant associations between the level of signal discrimination measured by the CPT, specifically on the degraded-stimulus CPT, and TDI factor scores for Fluid Thinking and Associative Disorganization during the inpatient period. They also observed that the outpatient signal discrimination deficits were significantly correlated with inpatient schizophrenic modes of thinking measured by the Rorschach Thought Disorder Index and with formal thought disorder measured by the BPRS Conceptual Disorganization rating.

Xu et al*.* [35] reported in a 1 year prospective study of language disorganization in patients with first-episode schizophrenia-spectrum disorders and investigated executive functions as a predictor of persistent FTD. They investigated the FTD using the Clinical Language Disorder Rating Scale (CLANG) [46] subdividing language abnormalities into syntactic, semantic, and production levels. They found that poorer performances in sustained attention and attention allocation/planning at illness onset were associated with an increased risk of having residual levels of semantic disorganization of language after 1 year, whereas poorer sustained attention was associated with increased risk of residual production problems. Pawelcyk et al*.* [47] evaluated pragmatic language functions in patients with first-episode psychosis, parents of the patients and healthy controls. Their results showed that the assessed groups varied in their ability to comprehend implicit information and to understand emotional prosody, as well as in processing language information regarding general knowledge, and in the effectiveness of interpersonal communication. More specifically, patients with first-episode psychosis performed significantly worse than healthy controls in all these neurocognitive domains. On the other hand, they found that the assessed groups did not differ regarding humour comprehension, understanding of linguistic prosody, understanding of both written and picture metaphors, or in their ability to process language information in the context of oral messages.

Caplan et al*.* [48] examined children with psychosis and investigated their use of discourse devices as well as the relation between FTD assessed with the K-FTDS and discourse deficits. They reported that patients not showing loose of associations used fewer words and conjunctions than controls. Furthermore, they observed that in the patients with loose of associations lexical cohesion correlated negatively with the total Intelligence Quotient (IQ) score, and with performance IQ, but not with verbal IQ. Remberk et al*.* [49] assessed the association between FTD, assessed through the TLC scale, and several neurocognitive domains in inpatients with early-onset schizophrenia-spectrum disorder and matched healthy controls. They assessed associations between psychopathological symptoms, cognitive functions, and FTD. The study showed that in patients FTD severity positively correlated with the number of non-perseverative errors on the Wisconsin Card Sorting Test (WCST), and with disturbances in semantic verbal fluency.

Ilonen et al*.* [34] explored a sample composed of clinical high-risk individuals (CHR), patients with psychosis and non-psychotic/non-CHR individuals. All individuals were administered a neuropsychological battery investigating verbal comprehension, perceptual organisation, working memory and processing speed, as well as measures of executive function and perceptual and thinking accuracy. They tested whether patients with CHR can be distinguished from psychotic and non-psychotic/non-CHR individuals using neuropsychological tests. They found that adolescents with CHR displayed poorer visual form perception and thinking disorder compared to non-psychotic/non-CHR individuals. They reported that the deficits observed in CHR were comparable in severity to those observed in adolescents with psychotic diagnoses and that patients with CHR displayed mild-to-moderate executive impairment, but no impairment in intellectual functioning.

## Discussion

Our literature survey showed that there was an increased interest for FTD in the early stages of psychosis from the late 70 s to late 80 s. Harrow et al*.* investigated in a few studies the course of psychosis after the first episode with a focus on FTD and formalized the following questions already in 1986 [32]: “Is thought disorder a frequent characteristic of acute schizophrenia? Does it persist in some or many patients with schizophrenia after the acute phase? And is it linked to other aspects of psychopathology? Does the presence of thought disorder after the acute phase predict subsequent clinical course and outcome?” These important questions reflect the core motivation for this systematic review covering the second half of the last century. Still, not all these questions have been answered. Even though the clinical importance of FTD has been observed from famous psychiatrists such as Kraepelin, Schneider, Bleuler, in the first half of the twentieth century, not so many studies in high-risk or stages of psychosis have been published due to methodological and phenomenological difficulties in the assessment of FTD with its multidimensional construct. [1, 2, 11, 12] Of note, early intellectual efforts on prevention and early diagnostics in psychiatry were started to be prominent only in the last 3 decades. Furthermore, the various measures of FTD among the included studies make a comparative interpretation of the associations between FTD and functioning and neurocognition difficult. Nevertheless, our review showed that FTD aspects linked to disorganization seem to be the most prominent in early psychosis, because of their consistent and replicated associations with both functioning and neurocognition aspects. Therefore, on the one hand, findings from this systematic review indicated that FTD, especially disorganization, might potentially have an early diagnostic and prognostic relevance in psychotic disorders, as (i) significant associations with functioning and cognition have been reported in both first-episode and clinical high-risk cohorts, and (ii) significant associations have been found in several subdomains of the functioning and neurocognition construct, spanning from general, to social, to role functioning, and hospitalization rate, for what concerns functioning, and from attention, executive function, and verbal IQ, for what concerns neurocognition. On the other hand, the predictive and generalizability potential of such symptoms in terms of disease and risk trajectories has not been fully explored yet in the early stages of psychotic disorders, given the paucity of studies included. In light of these few, but consistent associations across studies, we think that findings from this review suggest the urgency of spending more efforts in understanding the role of FTD into the risk pathways of psychosis. This is further testified by recent studies showing that FTD dimensions affect 55% of those presenting with first-episode psychosis and are associated with acute clinical presentation, poor quality of life and worse therapeutic relationships. [19, 50, 51] Furthermore, FTD were associated positively with patients’ unemployment risk [30], and negatively with their perceived quality of life [31] and overall life adjustment, based on indexes like work functioning, life disruptions and self-support. [30].

Moreover, given the significant associations between FTD, neurocognition and functioning impairments here reported, and given that these impairments very often precede the onset of full-blown psychosis and persist after the acute phase is resolved [24, 25], we speculate that FTD should be considered not only as a core psychosis characteristic but also as a feature of key importance to be targeted in early identification and intervention programs [19, 32]. This is further testified by the fact that six of the excluded studies (listed in Supplementary Table 1), although out of the review scope and thus not included in the “Results”, highlighted significant associations between FTD and transition to psychosis in high-risk groups. Indeed, Bearden et al*.* [44] reported that illogical thinking, POC, and referential cohesion, distinguished putatively CHR individuals who transited to psychosis from those who did not transit. Similarly, Demjaha et al*.* [52] found that scores on the negative and on the disorganization/cognitive dimensions in the Comprehensive Assessment of At-Risk Mental States (CAARMS) were associated with a transition to psychosis during the 24-month-follow-up in an at-risk mental state (ARMS)-cohort. Thompson et al*.* [53] found that the presence of FTD, especially thought blocking and tangentiality, together with elevated mood, predicted transition to psychosis. Consistently, Devlyder et al*.* [54] found that disorganized communication (i.e., subthreshold thought disorder) was associated with an increased hazard for psychosis onset, both at baseline and as a trajectory of high persistent disorganized communication. Katsure et al*.* [55] found that converter CHR showed more severe symptom scores for unusual thought content, disorganized speech, and emotional disturbances items, compared to non-converter CHR. Mamah et al. [56] found a significant association between disorganized communication and associated with psychosis conversion. Finally, Brucato et al*.* [57] showed that SIPS/SOPS Unusual Thought Content and Disorganized Communication subscales, measures of attenuated odd delusions and thought disorder, were very good predictors of psychosis, whereas attenuated suspiciousness and perceptual abnormalities were not associated with conversion.

Consistently with findings from our review, disorganization seems to be the most notable FTD symptom predicting transition to psychosis in high-risk groups. The POC was another FTD symptom that commonly associated with social and role functioning as well as with transition to psychosis. Not only these findings are consistent with the associations between FTD, functioning and neurocognition we have discussed, but they also further speak in favor of the clinical relevance of FTD in the pathophysiology of psychosis and encourage future research to longitudinally investigate their role in the clinical trajectories of individuals at-risk for psychosis. A potential way to methodologically accomplish this future research direction could be represented by further efforts in designing computational methods that might help clinicians in evaluating risk categories of patients at their first presentation also through the presence of FTD, especially in the disorganization domain. For example, some machine learning and computational pattern recognition studies provided promising results in automated computational speech or text analyses to improve the clinical utility and objective quantifiability of FTD. The earlier effort has appeared already in 2009 in the literature: Strous et al*.* [58] showed that patients with schizophrenia could be classified from non-affected individuals with 83.3% accuracy based on written text characteristics. Consistently, Bedi et al*.* [59] reported that speech features derived from a latent semantic analysis could successfully predict subsequent transition to psychosis (followed up quarterly for up to 2.5 years) in CHR. Other findings from the same group showed that an automated machine learning speech classifier could discriminate the speech of recent-onset psychosis patients from that of healthy individuals with a cross-validated predictive accuracy of 79%. [60] Furthermore, Mota et al*.* [61] showed that speech disorganization measured by graph connectedness could correctly predict schizophrenia diagnosis at 6-month follow-up with 91.67% accuracy in patients undergoing first clinical contact for recent-onset psychosis and 21 well-matched healthy subjects. Despite promising, these findings need to be externally replicated in greater samples to fulfil the challenging state-of-art criterion of machine learning applications in computational psychiatry [62].

Nevertheless, overall, these finding and those from our systematic review would support the relevance of FTD for several psychosis-associated outcomes, from neurocognition, to functioning, to transition to psychosis, and that applying cutting-edge methodological techniques to further FTD investigations may be a promising way to deliver clinicians with time-efficient and objective clinical tools supporting diagnostic and prognostic procedures. Many more future studies are warranted.

## Limitations

This review has some limitation. Mainly, studies have employed different assessment strategies to evaluate FTD, neurocognition and functioning. As a matter of fact, this high variability did not allow us to conduct any direct comparative analysis. Therefore, the results in this review can be only partially interpreted. We also acknowledge that our results are limited only to the published studies and that there was no study excluded due to non-significant associations. Therefore, a possible bias through studies showing non-significant associations between FTD, neurocognition and functioning could not be excluded.

## Conclusion

The reviewed studies showed that FTD severity is significantly associated with poor social functioning, unemployment, relapses of psychosis and re-hospitalisations, as well as transition to psychosis in high-risk groups. The results also showed significant associations between FTD and attention performance, executive functions, and verbal abilities. Machine learning algorithms show good potential for understanding the prognostic value of FTD in the risk trajectories of psychosis and encourage the development of computer-assisted early diagnostic tools targeting FTD, especially disorganization. Further studies taking advantage of the acceleration in computational psychiatry using methods such as unsupervised, supervised machine learning algorithms, deep learning techniques, natural language processing, sound, and rhythm analyses in records of patients’ clinical evaluations, would hopefully allow researchers to develop novel clinical markers for FTD. Such automated and time friendly computer-assisted diagnostic tools could give researchers and clinicians the chance to re-investigate the clinical importance of FTD starting from high risk and early stage of psychosis, and to fill the gap in the literature that might open new avenues to develop targeted neuropsychotherapeutics specific to FTD.

## Supplementary Information

Below is the link to the electronic supplementary material.

Supplementary file1 (DOCX 16 KB)
