# Supplementary material for: Association between formal thought disorders, neurocognition and functioning in the early stages of psychosis: a systematic review of the last half-century studies
Source: Eur Arch Psychiatry Clin Neurosci. 2021 Jul 14;272(3):381–93. doi: 10.1007/s00406-021-01295-3 (PMC8938342; doi:10.1007/s00406-021-01295-3)
Supplement: Supplementary file 1 — Supplementary file1 (DOCX 16 KB) [file 406_2021_1295_MOESM1_ESM.docx]

| **First Author** | **Publication Year** | **Published Journal** | **Study groups** | **Sample size** | **Mean age** | **FTD measures** | **Outcome measures** | **Main Findings & Conclusion** |
| --- | --- | --- | --- | --- | --- | --- | --- | --- |
| Bearden, CE | 2011 | Journal of the American Academy of Child & Adolescent Psychiatry | CHR | 105 | 16.66 | illogical thinking, poverty of content (POC), and referential cohesion | transition to psychosis | Transited to psychosis, predicted significantly social and role functioning at follow-up. |
| Demjaha, A | 2012 | Schizophrenia Bulletin | the ARMS | 122 | 23.4 | negative and disorganization/cognitive dimensions in the CAARMS | transition to psychosis | Formal thought disorder,  subjective cognitive impairments and negative symptoms are linked to the subsequent onset of psychosis. |
| Thompson, A | 2013 | Australian and New Zealand  Journal of Psychiatry | UHR | 120 | 18.3 | thought disorder assessed with the OPCRIT tool | transition to psychosis | The presence of formal thought disorder was most predictive when other symptoms were adjusted for. |
| DeVylder, JE | 2014 | Schizophrenia Research | CHR | 100 | 20.1 | SIPS/SOPS | transition to psychosis | Disorganized communication presented an increased hazard for psychosis onset. |
| Katsura, M | 2014 | Schizophrenia Research | the ARMS | 106 | 20 | CAARMS | transition to psychosis | Converters showed more severe symptom scores for the “unusual thought content,” “disorganized speech,” and “emotional disturbance” compared to non-converters. |
| Mamah, D | 2016 | Schizophrenia Research | HR  LR | 135  142 | 17.24 | SIPS, the WERCAP screen | transition to psychosis | Only disorganized communication as associated with psychosis conversion. |
| Brucato, G | 2017 | Psychological Medicine | APS, APSS | 200 | 20.4 | SIPS/SOPS, Kiddie Schedule for Affective Disorders and Schizophrenia--Present and Lifetime Version | transition to psychosis | Measures of attenuated odd delusions and thought disorder, best-predicted psychosis, |

**Supplementary Table 1. A list of studies for associations between FTD and transition to psychosis.**

FTD: Formal Thought Disorder, CDI: Communication Disturbances Index, CLR: Clinical Low Risk, CHR: Clinical High Risk, UHR: Ultra High Risk,

HR: high-risk, LR: low-risk, ARMS: At-Risk Mental State, APS: Attenuated Psychosis Syndrome, APSS: Attenuated Positive Symptom Syndrome,

OPCRIT: Operational Criteria for Psychotic Illness tool, CAARMS: Comprehensive Assessment of At-Risk Mental States, SIPS: Structured Interview

for Prodromal Syndromes, SOPS: Scale of Prodromal Symptoms, WERCAP: Washington early recognition center affectivity and psychosis screen
